# Epidemiology of CTX-M-type extended-spectrum beta-lactamase (ESBL)-producing nosocomial -*Escherichia coli* infection in China

**DOI:** 10.1186/s12941-015-0063-7

**Published:** 2015-01-16

**Authors:** Huiqing Shi, Fengjun Sun, Jianhong Chen, Qianyi Ou, Wei Feng, Xiaolan Yong, Peiyuan Xia

**Affiliations:** Department of Pharmacy, Southwest Hospital, Third Military Medical University, Chongqing, 400038 China; Department of Clinical Pharmacy, General Hospital of Chengdu Military Region, Chengdu, Sichuan Province 610083 China

**Keywords:** *Escherichia coli*, Epidemiology, Extended-spectrum beta-lactamase, Multilocus sequence typing, Nosocomial infection

## Abstract

**Background:**

*Escherichia coli* is one of the most common clinical pathogens causing nosocomial infection. The widespread cefotaxime-beta lactamases (CTX) has increased the multidrug resistance (MDR) of *E. coli* and has brought great trouble to the doctor treating the infection.

**Methods:**

ESBL-positive *E. coli* isolates were collected from different hospitals in different areas and the minimal inhibitory concentration (MIC) was analyzed by the agar dilution method. The resistance gene types were detected using polymerase chain reaction (PCR) and the sequence types were determined by multilocus sequence typing (MLST).

**Results:**

We found that the blaCTX-M-1 group and the blaCTX-M-9 group were the main CTX-M gene types, with many kinds of MLST gene types. Except for TEM with high isolate, SHV, OXA and VEB were relatively rare, while no PER and GES was detected. Most strains may have other resistance mechanisms, and the ESBL positive strains have high resistance not only to cephalosporins but also to other kinds of antibiotics.

**Conclusion:**

The study provides wide epidemiological data and enables more effective infection control and treatment plans.

## Introduction

*Escherichia coli* is one of the most common clinical pathogens causing nosocomial infection. For a long time, the widespread use of antibiotics to treat *E. coli* infectious disease has rapidly increased the multidrug resistance (MDR) of *E. coli* and hence has brought great trouble to the doctor treating the infection [1,2], especially with those strains producing extended-spectrum β-lactamase (ESBL). Although the Clinical and Laboratory Standards Institute (CLSI) reduced the necessity of ESBL screening and confirmatory tests and routine ESBL testing was no longer necessary in determining the dosage of antibiotics, ESBL testing is still useful for epidemiological purposes, because the ESBL-resistance gene carries a plasmid and transmits rapidly. After SHV-1 and TEM-1, the ESBL family of cefotaxime-beta lactamases (CTX) has been reported in the literature to be increasing in frequency around the world [3,4]. CTX-M-β-lactamases can be divided into five groups according to their amino acid sequence identities. Different CTX genotypes have different hydrolysis reactions to β-lactam antibiotics [5,6].

Obtaining accurate and prompt epidemiological data from CTX-M and other ESBL positive *E. coli* infections can enable an effective empirical therapy plan and infection control program. However, there has been little research in this area in China. In this study, we collected ESBL-positive *E. coli* from -different hospitals in different areas and analyzed the CTX-M and other ESBL gene type strains in China.

## Materials and methods

### Bacterial strains

A total of 342 isolated strains were collected from five general teaching hospitals in Chongqing, Henan, Tianjin, Hainan and Hebei from January 2012 to December 2013. Only samples collected from infectedsites of inpatients of more than 48 h were included in the study. The sources of the clinical specimens were as follows: urine (107), sputum (65), blood (52), pus (34), abdominal fluid (22), bile (15), wound (12), skin (9), pleural fluid (6), vagina (5), joint (5), catheter (4), cerebrospinal fluid (3), drainage liquid (2) and paracentesis fluid (1). The isolated strains were tested using biochemical assays, the Vitek system (bioMe’rieux Vitek) and conventional biochemical and growth methods. The presence of ESBLs was evaluated in both the control strains and the recent clinical isolates. Double-disk diffusion was used to detect ESBL production. The cefotaxime (CTX) and ceftazidime (CAZ) disks in combination with clavulanate (CLA) were performed and interpreted by CLSI criteria for ESBL screening and disk confirmation tests [7]. *Klebsiella pneumoniae* ATCC 700603 and *E. coli* ATCC 25922 were used as positive and negative controls, respectively. The non-repeated ESBL positive *E. coli* were collected, and all the strains were collected from different patients.

### Antimicrobial susceptibility

Antimicrobial susceptibility to a variety of drugs (including ampicillin, piperacillin, cefotaxime, cefepime, cefuroxime, cefoxitin, ceftazidime, aztreonam, imipenem, meropenem, ciprofloxacin, levofloxacin, gentamicin, amikacin, amoxicillin/clavulanic acid, ampicillin/sulbactam, piperacillin/tazobactam and trimethoprim-sulfamethoxazole) was evaluated by the agar dilution method according to CLSI guidelines (CLSI, 2014). *E. coli* ATCC 25922 and *Pseudomonas aeruginosa* ATCC 27853 were used as quality control strains.

### Molecular detection of ESBL

All the isolates were screened by PCR using the specific primers for each couple primer in Table 1 [8,9]. The PCR products were sent for DNA sequencing and the sequence results were analyzed using the NCBI BLAST program (http://www.ncbi.nlm.nih.gov/).

**Table 1:** **Primers used to amplify ESBL resistance genes**

**Target(s)**	**Sequence**
CTX-M group I^a^	GACGATGTCACTGGCTGAGC
	AGCCGCCGACGCTAATACA
CTX-M group II^b^	GCGACCTGGTTAACTACAATCC
	CGGTAGTATTGCCCTTAAGCC
CTX-M group III^c^	CGCTTTGCCATGTGCAGCACC
	GCTCAGTACGATCGAGCC
CTX-M group IV^d^	GCTGGAGAAAAGCAGCGGAG
	GTAAGCTGACGCAACGTCTG
CTX-M group V^e^	GCACGATGACATTCGGG
	AACCCACGATGTGGGTAGC
TEM	CATTTCCGTGTCGCCCTTATTC
	CGTTCATCCATAGTTGCCTGAC
SHV	AGCCGCTTGAGCAAATTAAAC
	ATCCCGCAGATAAATCACCAC
OXA^f^	GGCACCAGATTCAACTTTCAAG
	GACCCCAAGTTTCCTGTAAGTG
VEB	CATTTCCCGATGCAAAGCGT
	CGAAGTTTCTTTGGACTCTG
PER	GCTCCGATAATGAAAGCGT
	TTCGGCTTGACTCGGCTGA
GES	AGTCGGCTAGACCGGAAAG
	TTTGTCCGTGCTCAGGAT

^a^CTX-M-1, −3, −10 to −12, −15 (UOE-1), −22, −23, −28, −29, and −30.

^b^CTX-M-2, −4 to −7, and −20 and Toho-1.

^c^CTX-M-8.

^d^CTX-M-9, −13, −14, −16 to −19 and −21, and −27 and Toho-2.

^e^CTX-M-25 and −26.

^f^OXA-1.

### MLST analysis

Multilocus sequence typing (MLST) was performed for the *E. coli* isolates [8]. The primers of seven housekeeping genes, including adk, fumC, gyrB, icd, mdh, purA and recA, were PCR-amplified, purified and sequenced. The sequences were then compared with the PubMLST database http://mlst.warwick.ac.uk/mlst/dbs/Ecoli), and each unique combination of alleles (the allelic profile) was designated as a sequence type (ST) [10].

### Statistical analysis

The qualitative variables of the resistance rate to antimicrobial agents were compared using the chi-square test or the Fisher’s exact test with SPSS 10.0 software package. P ≤ 0.05 was considered statistically significant.

## Results

During the study period, we obtained 130 ESBL-producing strains from clinical specimens. Among the strains, 21 were from Hebei, 19 from Hainan, 30 from Henan, 30 from Tianjin and 30 from Chongqing. The sources of the clinical specimens were as follows: − urine (36), blood (21), sputum (20), pus (18), abdominal fluid (10), bile (8), wound (7), skin (5), vagina (2), pleural fluid (2) and joint (1). The resistance rate of ESBL-producing *E. coli* from different areas is presented in Table 2. The ESBL positive strains were highly resistant to cephalosporins and fluoroquinolones. However, the strains responded better to the enzyme inhibitor and β-lactam antibiotics. Only three strains were resistant to imipenem, and two were resistant to meropenem. The cephalosporins and fluoroquinolones resistance rate of isolated strains from Hainan was lower than that of the strains from the other hospitals.

**Table 2:** **Antimicrobial susceptibility of ESBL-producing** ***Escherichia coli*** **isolates from different areas**

**Antimicrobial agent**	**Hainan (n = 19)**	**Hebei (n = 21)**	**Chongqing (n = 30)**	**Henan (n = 30)**	**Tianjin (n = 30)**
Ampicillin	100	100	100	100	100
Piperacillin	100	100	100	100	93.3
Cefotaxime	94.7	100	100	100	90
Cefepime	68.4	95.2*	93.3	90	83.3
Cefuroxime	100	100	100	100	96.7
Cefoxitin	15.8	23.8	36.7	16.7	20
Ceftazidime	26.3	61.9*	56.7*	63.3*	53.3
Aztreonam	5.3	61.9**	73.3**	76.7**	60**
Imipenem	0	0	3.3	6.7	0
Meropenem	0	0	6.7	0	0
Amoxicillin/Clavulanic acid	84.2	9.5**	43.3**	50*	20**
Ampicillin/Sulbactam	73.7	71.4	80	83.3	76.7
Piperacillin/Tazobactam	5.3	0	23.3	3.3	6.7
Trimethoprim-sulfamethoxazole	78.9	85.7	83.3	83.3	73.3
Ciprofloxacin	68.4	90.5	70	76.7	60
Levofloxacin	47.4	90.5**	70	76.7*	60
Amikacin	5.3	0	16.7	6.7	6.7
Gentamycin	52.6	85.7*	63.3	66.7	76.7

**P < 0.01 and *P < 0.05.

There were 126 (96.9%) ESBL-producing isolates of amplified blaCTX-M genes, while only four strains were CTX-M negative. The positive rate of the ESBL resistance genes from different areas is presented in Table 3. The blaCTX-M-1 group and the blaCTX-M-9 group were detected at 66.9% (87/130) and 54.6% (71/130), respectively. No blaCTX-M-2, blaCTX-M-8, and blaCTX-M-25 groups were detected in the isolates. The blaCTX-M-9 group had CTX-M-65 and CTX-M-14. The blaCTX-M-1 group had CTX-M-3 and CTX-M-15. There were 110 (84.6%) strains of amplified TEM. The OXA and SHV genes were detected at 21.5% (28/130) and 17.7% (23/130), respectively. Only 7 isolates were VEB positive. Neither PER nor GES was detected in the strains. Most strains had more than two resistance genes.

**Table 3:** **Distribution of ESBL resistance genes in different areas**

	**Hainan (n = 19)**	**Hebei (n = 21)**	**Chongqing (n = 30)**	**Henan (n = 30)**	**Tianjin (n = 30)**
blaCTX-M-1 group	57.9(11)	42.9(9)	83.3(25)	83.3(25)	56.7(17)
blaCTX-M-9 group	68.4(13)	71.4(15)	53.3(16)	26.7(8)	63.3(19)
TEM	63.2(12)	85.7(18)	96.7(29)	83.3(25)	86.7(26)
SHV	0(0)	14.3(3)	30.0(9)	13.3(4)	23.3(7)
OXA	10.5(2)	33.3(7)	26.7(8)	16.7(5)	20.0(6)
VEB	0(0)	0(0)	16.7(5)	0(0)	6.7(2)

MLST results showed that 130 isolated strains had 42 gene types. The ST131 and ST167 were the most common genotypes with 19 and 12 strains separately. ST38, ST10, ST405 and ST2003 were less common with 9, 7, 7, and 6 isolates separately. For others, there were two genotypes having 5 strains, three having 4 strains, four having 3 strains, 9 having 2 strains and 18 having only 1 strain.

## Discussion

This study presented the epidemiology of CTX-M *E. coli* from five hospitals in different cities of China. The results showed that the ESBL-producing isolates were highly resistant to both the cephalosporins and the fluoroquinolones confirming other reports [8,11]. This suggests that the ESBL resistance gene may exist with other resistance genes. Hence cephalosporin and fluoroquinolone are not considered to be effective choices for treatment of patients with ESBL-producing Enterobacteriaceae infection because of their relatively high resistance rates. However, the resistance rates of ceftazidime and aztreonam from Hainan hospital were lower than those from the other hospitals and other reports [8,12,13]. This demonstrates that other resistance mechanisms exist along with the ESBL in most strains. In our study, only four carbapenem-resistant strains were detected, so carbapenems were recommended as reserve antimicrobials to treat ESBL bacterial infections. Though the five hospitals were widely separated in China, the resistance rate of clinical isolates from the hospitals to the tested drugs was similar except the strains from Hainan, which showed that the ESBL-producing strains had a similar drug resistance spectrum.

All ESBL-positive strains from the five hospitals were analyzed for blaCTX-M genes by PCR assay. The results showed that almost all isolates (126/130) - carried blaCTX-M. Obviously, the blaCTX-M type is the most common ESBL resistance gene in China, as most reports indicate. The CTX-M ESBL *E. coli* positive rate is higher than that reported abroad [8,14,15], which is due to the wide use of - β-lactam antibiotics. At the same time, the main CTX-M gene types of the five cities were CTX-M-14 and CTX-M-15, which is confirmed by most research in China [8,16]. At the same time, the positive rates of the blaCTX-M-1 group and the blaCTX-M-9 group have some differences, which suggest that there are regional epidemiological characteristics. This result suggests that CTX-M-14 is widely distributed among hospital infection patients, and that when doctors doubt whether ESBL *E. coli* infection is present, they try to avoid using the β-lactam antibiotics. The β-lactam/β-lactamase inhibitor combinations, carbapenems and amikacin should first be considered. The results for the TEM, SHV, OXA, VEB, PER and GES genes assay showed that TEM-positive *E coli* strains were also very common in the five hospitals, while the others were relatively rare, which was consistent with the results in previous studies [17,18]. At the same time, the positive rates of the TEM, SHV, OXA and VEB gene have some differences among areas, indicating that there are some regional epidemiological characteristics. The study showed that the CTX-M-type ESBL was the most common resistance gene type. As the different ESBL resistance genes have different hydrolysis capabilities with differentβ-lactams antibacterials, so the different ESBL distributions should be considered in antibacterial use.

All the clinically isolated strains belonged to 42 gene types. ST131 was the most common with 19 strains, which was confirmed by domestic and international research [8,19-21]. ST167 was the second with 12 strains. ST167 was also a common genotype in *E coli* in foreign research [22], which was seldom reported in China [23]. ST167 was listed a lineage with a potential extended host spectrum genotype. Except for ST131 and ST167, no other types had more than 10 strains. There was no obvious relationship between the MLST gene type and ESBL resistance gene type. This result suggests that the ESBL gene is widely distributed in different MLST gene types, which is confirmed by other reports.

This research has two limitations: first, the clinical information was not acquired, and we could not further analyze the risk factors for ESBL *E. coli* infections; second, the number of strains was not large enough to display epidemiological features. Generally speaking, the study examined the drug resistance and genotypic epidemiology of CTX-M type clinically isolated *E. coli*. We found that the CTX-M is still the primary genotype of ESBL in China, and that the ESBL positive strains have higher resistance. The CTX-M-14 and CTX-M-15 are the most common resistance gene types and ST131 is the predominant clonal group. This study will provide reference data to enable relevant infection control and treatment.
